# Verbal Memory Impairment in Patients with Subsyndromal Bipolar Disorder

**DOI:** 10.3389/fpsyt.2017.00168

**Published:** 2017-09-15

**Authors:** Tomiki Sumiyoshi, Atsuhito Toyomaki, Naoko Kawano, Tomoko Kitajima, Ichiro Kusumi, Norio Ozaki, Nakao Iwata, Kazuki Sueyoshi, Kazuyuki Nakagome

**Affiliations:** ^1^Department of Clinical Epidemiology, Translational Medical Center, National Center of Neurology and Psychiatry, Tokyo, Japan; ^2^Department of Neuropsychiatry, Graduate School of Medicine, Hokkaido University, Sapporo, Japan; ^3^Department of Psychiatry, Nagoya University Graduate School of Medicine, Nagoya, Japan; ^4^Department of Psychiatry, Fujita Health University School of Medicine, Toyoake, Japan; ^5^National Institute of Health, National Center of Neurology and Psychiatry, Tokyo, Japan

**Keywords:** bipolar disorder, verbal learning, California Verbal Learning Test-II, HVLT-R, Brief Assessment of Cognition in Schizophrenia

## Abstract

**Backgrounds:**

Several domains of cognitive function, including learning memory and executive function, are impaired in mood disorders. Also, the relationship between disturbances of these two cognitive domains has been suggested. In line with the recent initiative to establish a standard measure of cognitive decline in bipolar disorder, the present study was conducted to (1) test the criterion-related validity and test–retest reliability of the California Verbal Learning Test (CVLT)-II Japanese version, and (2) determine if type of word learning tasks (i.e., with or without a category structure) affects severity of verbal memory deficits in patients with subsyndromal bipolar disorder.

**Methods:**

Thirty-six patients with bipolar disorder with mild symptoms and 42 healthy volunteers participated in the study. We first compared effect sizes for memory deficits in patients among the CVLT-II, Brief Assessment of Cognition in Schizophrenia (BACS), and Hopkins Verbal Memory Tests-Revised (HVLT-R). We next evaluated the correlations between scores of the CVLT-II vs. those of the BACS and HVLT-R. Bipolar patients were re-assessed with the same (standard) or alternate forms of the CVLT-II and HVLT-R 1 month later.

**Results:**

Scores on the CVLT-II 1–5 Free Recall and Long-delay Free Recall, as well as the HVLT-R Immediate Recall, but not the BACS List Learning were significantly lower for patients compared to control subjects. The effect sizes for cognitive decline due to the illness were comparable when measured by the CVLT-II and HVLT-R, ranging from 0.5 to 0.6. CVLT-II scores were significantly correlated with those of the HVLT-R and BACS. Test–retest reliability of the CVLT-II was acceptable, and no significant practice effect was observed when the alternate form was used. There was no consistent relationship between mood symptoms and performance on the CVLT-II.

**Conclusion:**

These results suggest the CVLT-II Japanese version is able to discriminate between bipolar disorder patients and healthy controls with good sensitivity and validity. Data in this study also indicate that the degree of verbal memory deficits in bipolar disorder may be influenced by memory organizational strategy.

## Introduction

Bipolar disorder is associated with poor psychosocial outcome not only in the manic or depressive state, but also in the subsyndromal state (1–3). Patients with the illness demonstrate impairments in several cognitive domains even during the euthymic phase (4). The profile of cognitive disturbances of bipolar disorder has been reported to be similar to that of schizophrenia (5–7), with less severity. Thus, the effect size of euthymic patients ranges from 0.4 to 0.7 compared to healthy controls (8). Specifically, Martínez-Arán et al. (9) demonstrated that duration of illness, a history of psychotic symptoms, number of hospitalizations, manic episodes, and suicide attempts were positively related to cognitive impairments. Importantly, cognitive impairments have been suggested to predict poor psychosocial outcome in bipolar disorder patients (10–12).

There may be interactions in the disturbances of key cognitive domains in bipolar disorder. For example, the contribution of executive function to learning memory has been reported in patients with the disease (13). Specifically, Deckersbach et al. (13) report that verbal learning memory deficits are mediated by semantic clustering encoding (memory organization) strategies. This finding may be important in understanding the nature of cognitive impairment of mood disorders.

To evaluate verbal memory in subjects with bipolar disorder, several tasks, including the Brief Assessment of Cognition in Schizophrenia (BACS) (14), Hopkins Verbal Learning Test-Revised (HVLT-R) (15), and the California Verbal Learning Test (CVLT) (16, 17) have been used. For example, the CVLT has been recommended to assess verbal learning in bipolar disorder (16). In this line, the reliability of the CVLT-II has been reported to be acceptable with good internal consistency, whose normative data have been shown to be more representative of the general population than that for the CVLT (18).

The effect size of cognitive decline in euthymic patients ranges from 0.66 to 0.90 (19). As verbal (learning) memory provides one of the important domains of cognition in psychiatric diseases (20), it is worthwhile to explore which factors contribute to its impairment in bipolar patients whose mood symptoms are not so eminent. Since executive function, another pivotal domain of cognition related to frontal lobe function, is impaired in euthymic bipolar patients (21), it is hypothesized that verbal memory deficits become evident when assessed with word list tasks that require memory organizational strategy, but not those that do not require it.

The main purpose of this study was to investigate the impact of memory organizational strategies on verbal (learning) memory, as measured by performance on word list tasks, in patients with bipolar disorder. For this aim, we sought to determine whether the CVLT-II and HVLT-R, but not the BACS would be able to discriminate between patients with subsyndromal bipolar disorder and normal control subjects. This is based on the assumption that only the former two tasks require subjects to use memory organization. Additionally, we investigated the validity and reliability of the Japanese version of CVLT-II using the BACS List Learning and HVLT-R as reference measures of verbal learning and memory. Preliminary analyses of the present data have been reported (22, 23).

## Materials and Methods

### Subjects

This was a multi-center collaborative study, whose design, characteristics of participants, and other information have been registered (UMIN ID: 000013623). The sample consisted of 78 participants; 36 individuals with bipolar disorder and 42 healthy control participants who were native Japanese and had no history of psychiatric disorders (Table 1). Bipolar disorder patients were diagnosed by clinicians according to DSM-IV criteria and showed a subsyndromal or non-significant clinical level of severity of mood symptoms, i.e., ratings with the Montgomery–Asberg Depression Rating Scale (MADRS) ≤14 and the Young Mania Rating Scale (YMRS) ≤14 (24). The patients were recruited from the National Center of Neurology and Psychiatry Hospital, Fujita Health University Hospital, Nagoya University Hospital and Hokkaido University Hospital. Healthy volunteers as a control group were recruited from the local community. They were matched with bipolar disorder patients in terms of age and sex (Table 1). There were no between-group differences in educated years and premorbid IQ estimated using the Japanese Adult Reading Test (25). Patients with comorbid neurological illness, previous traumatic brain injury with any known cognitive consequences or loss of consciousness for more than 5 min, or alcohol/substance abuse or addiction (except nicotine) were excluded. Six patients had a history of suicide attempt and two received electroconvulsive therapy. The patients were taking lamotrigine (for 16 cases), lithium (15), aripiprazole (14), valproate (11), quetiapine (9), olanzapine (3), carbamazepine (2), risperidone (2), sertraline (2) and levomepromazine (2), chlorpromazine (1), duloxetine (1), and maprotiline (1).

**Table 1:** Clinical and demographic variables (mean ± SD).

	Bipolar disorder	Healthy controls
Sex (male:female)	12:24	17:25
Age (years)	39.2 ± 9.2	36.9 ± 10.0
Type (A-A:A-B)	17:19	18:24
Educated years	15.0 ± 2.4	14.1 ± 2.1
JART	107.0 ± 9.2	106.9 ± 8.2
MADRS	6.2 ± 4.4	–
YMRS	2.3 ± 3.1	–
Subtype (BP1:BP2)	9:27	–
Non-remission patients	9	–
History of psychosis	8	–
Duration of illness (months)	92.0 ± 1.1	–
Number of hospitalization	1.1 ± 1.6	–

*A-A, standard–standard; A-B, standard–alternate; JART, Japanese Adult Reading Test; MADRS, Montgomery–Asberg Depression Rating Scale; YMRS, Young Mania Rating Scale*.

Written consent was obtained from all participants, according to ethics guidelines set out by each participating site. The study protocol was approved by the ethics committees of participating institutions.

### The Study Design

The subjects were administered three verbal learning tests twice with an interval of approximately 1 month. Mood symptoms were also assessed each time using the MADRS and YMRS. The CVLT-II, BACS, and HVLT-R forms were switched to alternate forms in 43 subjects (from Nagoya University Hospital and Hokkaido University Hospital) at the follow-up (19 patients and 24 controls), whereas 35 subjects (from National Center of Neurology and Psychiatry Hospital and Fujita Health University Hospital) were administered the same standard form at the follow-up (17 patients and 18 controls) as at the baseline. The same raters performed these cognitive tests at baseline and the 1-month follow-up assessments.

### Development of the CVLT-II Japanese Version

To develop a Japanese version of the CVLT-II (26), one of the authors (Tomiki Sumiyoshi) translated the original CVLT-II from English to Japanese. Afterward, a person isolated from the translator performed a back-translation. Modifications of some terms were made to fit the local culture. The back-translation of the English version was approved by Pearson Education, Inc., the copyright owner.

### Measures of Verbal Learning

#### California Verbal Learning Test-II

The CVLT-II measures both recall and recognition abilities using two word lists. In the first five trials, immediately after presentation of List A, the subject is asked to recall the words of the list. List A contains 4 words from each of 4 semantic categories, for a total of 16 words. This procedure enables evaluation of semantic clustering ability, the most effective strategy for learning non-systemized verbal information. Subsequently, an interference list (List B) containing 16 words is presented, followed by a recall test. The interference test is followed by a short-delay free recall test and a short-delay cued recall test using List A. Then, following 20-min interval, a long-delay free recall test, long-delay cued recall test, and yes/no recognition test are administered using List A. After the yes/no recognition test, a new approximately 10-min forced-choice recognition test is arbitrarily administered. In the present study, data obtained in the immediate recall test after the first five trials of List A (“1–5 free recall”) and a long-delay free recall test were adopted for analyses, which were the candidate measures to be incorporated in the International Society for Bipolar Disorders-Battery for Assessment of Neurocognition (4). We basically intended to use standardized measure for all cognitive tests; however, since it is not available only for the CVLT-II (Japanese version), we used raw scores for this test.

#### HVLT-R

The HVLT-R consists of a word list, containing 3 words from one of 4 semantic categories, for a total of 12 words. In the first three trials, immediately after presentation of the word list, the subject is asked to recall the words of the list. Subsequently, following a 20–25-min interval, a delayed recall test is administered. Immediately after the delayed recall test, a forced-choice recognition test is administered. In the present study, the delayed recall test and forced-choice recognition test were not included for brevity, and only a standardized measure in the immediate recall test after the first three trials was adopted for analyses, which is used in the MATRICS Consensus Cognitive Battery (the standardized measure was obtained by using the mean level of 28.2 and the SD of 4.3).

#### Brief Assessment of Cognition in Schizophrenia

The BACS List Learning test consists of a word list, containing 15 words. The subject is asked to recall the words of the list immediately after presentation of the word list, which was repeated five times. The words in the list were not semantically organized unlike the case in the CVLT-II and HVLT-R. In the present study, a standardized measure in the immediate recall test after the first five trials was adopted for analyses. (The standardized measure was obtained by using the mean level of 49.2 and the standard deviation of 9.9.) (27).

### Statistical Analysis

Student’s *t*-test was performed to explore between-group differences for CVLT-II 1–5 free recall scores and long-delay free recall scores, HVLT-R immediate recall scores, and BACS list learning scores at baseline. If a significant between-group difference in either measure was found, the effect size was calculated using a Cohen’s *d* to explore its sensitivity. To examine the effect of mood symptoms, Spearman’s rank correlation was performed between CVLT-II 1–5 free recall or long-delay free recall scores vs. MADRS and YMRS scores. Test–retest reliability was evaluated using intraclass correlation coefficient (ICC) (28) between scores at baseline and 1-month follow-up for each measure. In addition, practice effect was evaluated using repeated measures ANOVA using “time” as an intra-individual factor and “Group” and “type (A-A, A-B)” as inter-individual factors. Secondary analyses were performed when a significant interaction between the factors was obtained.

To examine the criterion-related validity of the CVLT-II measures, Pearson’s product-moment correlation among scores of the CVLT-II 1–5 Free Recall and Long-Delay Free Recall, HVLT-R Immediate Recall, and BACS List Learning was calculated for patients.

## Results

### Between-Group Comparison of Verbal Learning Measures

**Figure 1:**
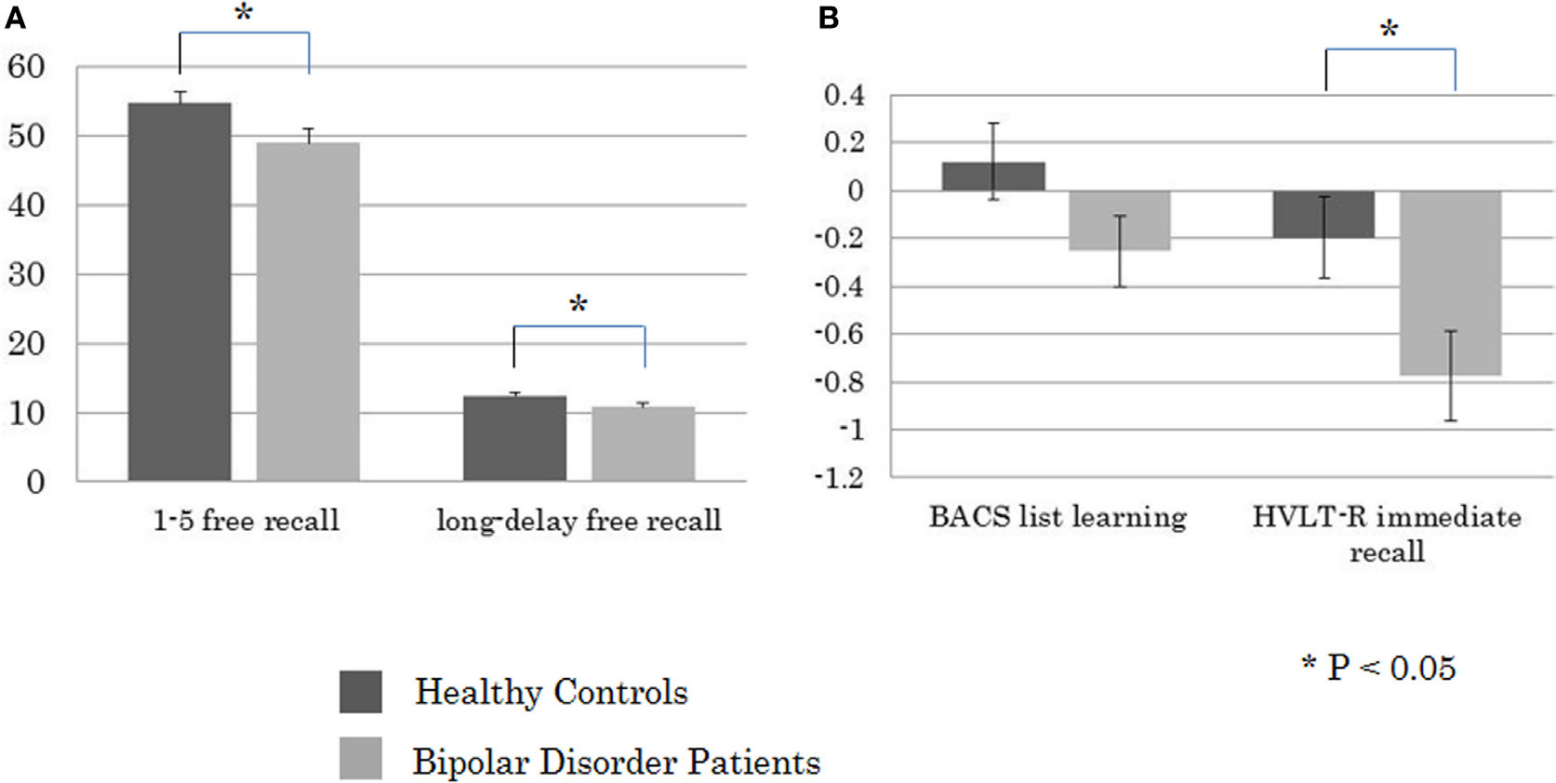
Between-group comparisons in performance on **(A)** the California Verbal Learning Test-II 1–5 free recall and long-delay free recall (presented in raw score), and **(B)** the Brief Assessment of Cognition in Schizophrenia (BACS) list learning and HVLT-R immediate recall (presented in *z*-score).

Student’s *t*-test revealed a significant between-group difference for the CVLT-II 1–5 Free Recall (*t* = −2.28, *P* = 0.025) and Long-Delay Free Recall (*t* = −2.04, *P* = 0.035), and HVLT-R Immediate Recall (*t* = −2.47, *P* = 0.016), but not the BACS List Learning (*t* = −1.58, n.s.) (Figure 1). The effect sizes of performance on the measures that showed between-group differences were 0.52, 0.46, and 0.56, respectively. Significant positive correlations were noted between ratings with the MADRS vs. scores of the CVLT-II 1–5 Free Recall (Rho = 0.36, *P* = 0.029), and Long-Delay Free Recall (Rho = 0.34, *P* = 0.044). On the other hand, performances on these CVLT measures were not correlated with YMRS scores (1–5 Free Recall, Rho = 0.12, n.s.; Long-Delay Free Recall, Rho = 0.03, n.s.).

### Test–Retest Reliability and Practice Effect of Verbal Learning Measures

The ICCs between the baseline and 1-month follow-up scores for the BACS List Learning, HVLT-R Immediate Recall, CVLT-II 1–5 Free Recall and Long-Delay Free Recall are summarized in Table 2. Good to excellent test–retest reliability was noted in most of the measures.

**Table 2:** Intraclass correlation coefficients between the baseline and 1-month follow-up scores.

		Bipolar disorder	Healthy controls
Brief Assessment of Cognition in Schizophrenia list learning		0.65	0.57
	A-A	0.84	0.52
	A-B	0.37	0.62

HVLT-R immediate recall		0.83	0.78
	A-A	0.88	0.82
	A-B	0.74	0.73

California Verbal Learning Test (CVLT)-II 1–5 free recall		0.62	0.65
	A-A	0.63	0.62
	A-B	0.64	0.70

CVLT-II long-delay free recall		0.67	0.63
	A-A	0.58	0.82
	A-B	0.79	0.52

*A-A, standard–standard; A-B, standard–alternate*.

### Practice Effects

**Figure 2:**
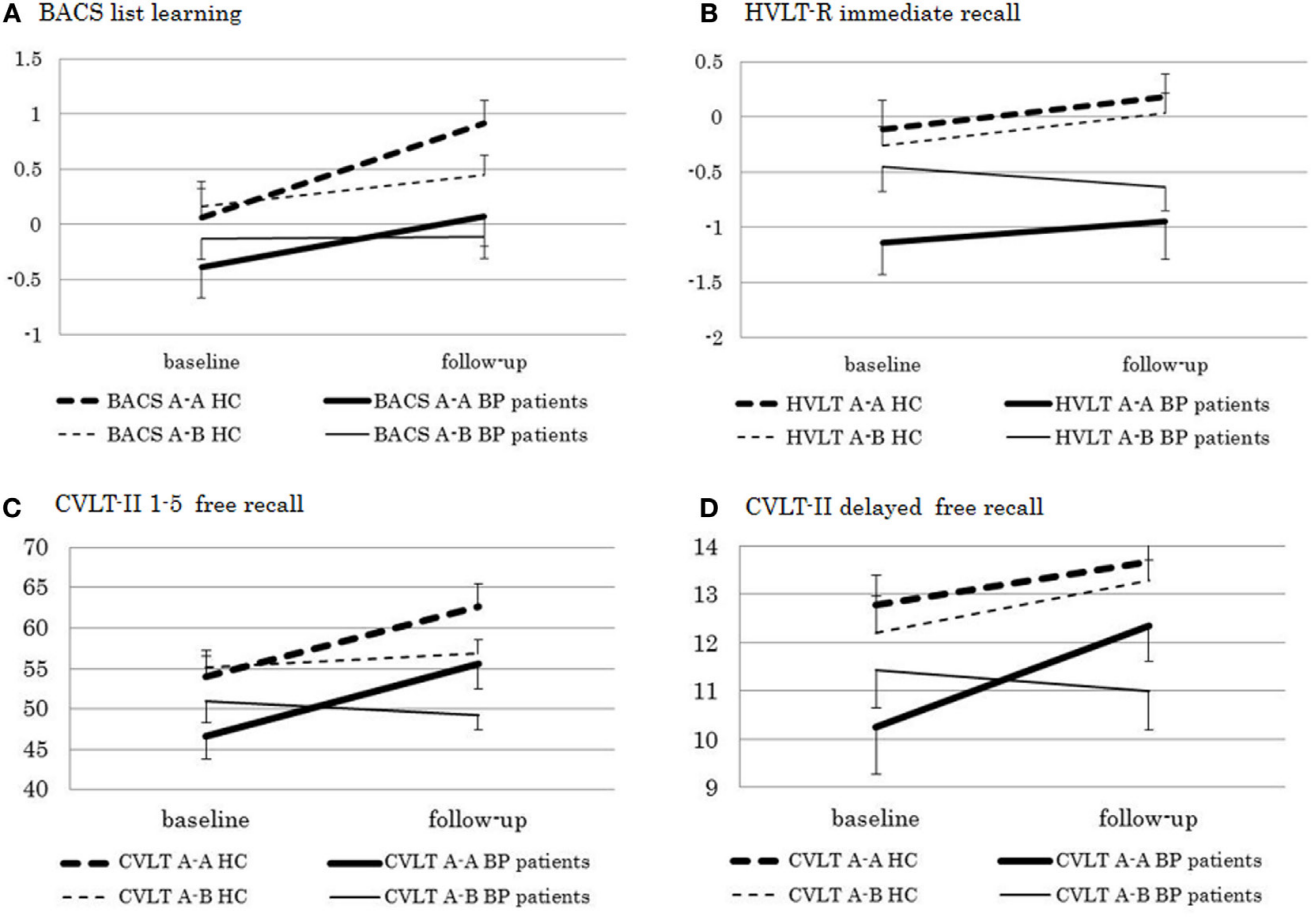
Test–retest performance on **(A)** the Brief Assessment of Cognition in Schizophrenia (BACS) list learning and **(B)** HVLT-R immediate free recall (presented in *z*-score), and **(C)** CVLT-II 1–5 free recall and **(D)** delayed free recall (presented in raw score) in bipolar disorder patients and healthy controls. A-A, standard form–standard form pattern; A-B, standard form–alternate form pattern.

A significant main effect of “group” (*F* [1, 74] = 6.58, *P* = 0.012) and “time” (*F* [1, 74] = 21.44, *P* < 0.0001) and a significant “type” × “time” interaction (*F* [1, 74] = 8.26, *P* = 0.005) were found on scores of the BACS List Learning (Figure 2). Accordingly, a secondary analysis was performed for each “type,” which revealed a significant main effect of “time” in type A-A (*F* [1, 33] = 49.60, *P* < 0.0001), but not type A-B (*F* [1, 41] = 1.23, n.s.). As for the HVLT-R, there was a significant main effect of “group” (*F* [1, 74] = 10.05, *P* = 0.002), while “time” effect did not reach a significant level (*F* [1, 74] = 3.94, *P* = 0.051). A secondary analysis for each “group” revealed no significant main effect of “time” or “time” × “type” interaction in either healthy controls (“time”: *F* [1, 40] = 0.84, n.s.; “time” × “type” interaction: *F* [1, 40] = 0.00, n.s.) or bipolar disorder patients (“time”: *F* [1, 34] = 2.56, n.s.; “time” × “type” interaction: *F* [1, 34] = 2.90, *P* = 0.097). Repeated measures ANOVA for CVLT-II 1–5 Free Recall revealed a significant effect of “group” (*F* [1, 74] = 8.24, *P* = 0.005) and “time” (*F* [1, 74] = 27.97, *P* < 0.0001). “Type” × “time” interaction was also significant (*F* [1, 74] = 28.42, *P* < 0.0001). Therefore, secondary analysis for each “type” was performed. There was a significant effect of “time” in type A-A (*F* [1, 33] = 67.71, *P* < 0.0001), but not type A-B (*F* [1, 41] = 0.00, n.s.). In addition, “group” × “time” interaction was not significant in either “type” (type A-A: *F* [1, 33] = 0.02, n.s.; type A-B: *F* [1, 41] = 1.94, n.s.). As for CVLT-II Long-Delay Free Recall, “group” effect (*F* [1, 74] = 6.76, *P* = 0.011) and “time” effect (*F* [1, 74] = 11.47, *P* = 0.001), as well as “type” × “time” interaction (*F* [1, 74] = 4.68, *P* = 0.034) were significant. Also, “group” × “type” × “time” interaction was significant (*F* [1, 74] = 6.37, *P* = 0.014). A secondary analysis revealed a significant effect of “time” in type A-A (*F* [1, 33] = 17.21, *P* = 0.0002), but not type A-B (*F* [1, 41] = 0.72, n.s.). Moreover, “group” × “time” interaction was not significant in either pattern (type A-A: *F* [1, 33] = 2.87, n.s.; type A-B: *F* [1, 41] = 3.72, n.s.).

### Criterion-Related Validity

The criterion-related validity of the CVLT-II 1–5 Free Recall and Long-Delay Free Recall tasks were examined using the HVLT-R Immediate Recall and BACS List Learning tasks in patients with bipolar disorder. Pearson’s product-moment correlation coefficients ranged from 0.68 to 0.81.

## Discussion

The CVLT-II Japanese version and HVLT-R, but not BACS were found to discriminate between bipolar disorder patients and healthy individuals with a sensitivity comparable to that of the HVLT-R. Strong correlations with performances on the BACS List Learning and HVLT-R Immediate Recall suggest a good criterion-related validity of the CVLT-II as a tool to detect cognitive disturbances in patients with bipolar disorder. There was no consistent relationship between mood symptoms and performance on the CVLT-II in the subsyndromal patients.

### Between-Group Differences

Interestingly, between-group differences were significant in scores of the CVLT-II 1–5 Free Recall and Long-Delay Free Recall, and HVLT-R Immediate Recall, but not BACS List Learning. One of the reasons may be that the word lists in the CVLT-II and HVLT-R are semantically organized while this is not the case with the BACS. It may be that bipolar disorder patients show impairment in semantic clustering, in agreement with previous suggestions that the impairment in verbal organizational strategies causes the difficulty in recalling words (13, 29).

The patient–control effect size of the CVLT-II 1–5 Free Recall (0.52) was smaller than those previously reported using the CVLT (0.73–0.82) (4). Meanwhile, the effect size for the HVLT-R Immediate Recall (0.56) was slightly larger than that reported in Schretlen et al. (30) for bipolar disorder patients (0.42) (30). The overall performance on the CVLT-II 1–5 Free Recall at baseline (0.73 for the healthy controls and 0.65 for the bipolar disorder patients) were still worse than that in the HVLT-R Immediate Recall (0.76 and 0.69), suggesting a satisfactory level of cognitive demands of the CVLT-II.

### Test–Retest Reliability and Practice Effect of Verbal Learning Measures

We found moderate to good test–retest reliability in both CVLT-II 1–5 Free Recall and Long-Delay Free Recall scores in bipolar disorder patients and healthy controls. In a previous investigation on the CVLT-II,^17^ the reliability coefficients ranged from 0.72 to 0.79 in a sample of 288 healthy subjects, with a median interval of 21 days, similar to the ICC for the CVLT-II 1–5 free recall in healthy controls using an alternate form (0.70) in the present study (Table 2). Interestingly, the HVLT-R Immediate Recall generally showed greater ICC values than the other tests, attaining a good to excellent level.

### Criterion-Related Validity

Both CVLT-II 1–5 Free Recall and Long-Delay Free Recall scores showed strong correlation with either the BACS List Learning or HVLT-R Immediate Recall scores, suggesting good criterion-related validity.

### Relationship of Performance on the CVLT-II with Mood Symptoms

Meta-analytic studies generally report that bipolar patients with the more severe depressive or manic symptoms are likely to show the worse performance on tests of learning and memory [reviewed in Ref. (19)]. On the other hand, the lack of a significant relationship between manic symptoms, measured by the YMRS, and performance on the CVLT-II, reported here, may be related to the inclusion of subsyndromal patients. The *positive* correlations between ratings with the MADRS and CVLT-II scores, obtained in this study, seem somewhat contradictory and might be also due to the nature of the subjects studied.

### Task-Specific Decline in Memory Performance in Bipolar Disorder

The CVLT-II is characterized by the (four category)/(four words per category) structure (26), while the HVLT-R consist of (three category)/(four words per category) (15). On the other hand, the BACS List Learning does not have such “internal category” structure. This difference may provide a major reason why only the former two tasks were able to discriminate between patients and control subjects. This concept may be partly supported by Deckersbach et al. (13), who found the contribution of memory organizational strategy to poor performance on the Long-Delay Free Recall Task of the CVLT. Further studies with data from other psychiatric conditions would help understand the nature of cognitive impairment of mood disorders.

### Limitations

Unlike previous studies, patients with bipolar disorder studied here were not necessarily in the *euthymic* state, although all met the *subsyndromal* state criterion. There is a possibility that the patient-control effect sizes may have been overestimated due to residual depressive symptoms. However, a *positive* correlation between rating with the MADRS vs. CVLT-II 1–5 Free Recall and Long-Delay Free Recall scores may argue against this view.

## Conclusion

The Japanese version of CVLT-II appeared to provide valid measures of verbal learning and memory function in bipolar disorder patients. The ability of the CVLT-II and HVLT-R, but not BACS List Learning, to discriminate between patients and control subjects may be related to the use of memory organization strategy specific to the CVLT-II and HVLT-R, which deserves further study.

## Ethics Statement

This study was carried out in accordance with the recommendations of “name of guidelines, name of committee” with written informed consent from all subjects. All subjects gave written informed consent in accordance with the Declaration of Helsinki. The protocol was approved by ethics committees of the National Center of Neurology and Psychiatry Hospital, Fujita Health University Hospital, Nagoya University Hospital, and Hokkaido University Hospital.

## Author Contributions

Contributions of each author are as follows: conception and design of the study (TS); acquisition of data (AT, NK, TK, IK, NO, and NI); analysis of data (KN and KS); and drafting of the manuscript (KN and TS).

## Conflict of Interest Statement

There are no conflicts of interest for any of the authors of this paper. No author has any possible financial gain for the findings presented here.
